# Diagnostic Value of Urinary Kidney Injury Molecule 1 for Acute Kidney Injury: A Meta-Analysis

**DOI:** 10.1371/journal.pone.0084131

**Published:** 2014-01-03

**Authors:** Xinghua Shao, Lei Tian, Weijia Xu, Zhen Zhang, Chunlin Wang, Chaojun Qi, Zhaohui Ni, Shan Mou

**Affiliations:** Department of Nephrology, Molecular Cell Lab for Kidney Disease, Ren Ji Hospital, School of Medicine, Shanghai Jiao Tong University, Shanghai, China; University Hospital Heidelberg, Germany

## Abstract

**Background:**

Urinary Kidney Injury Molecule 1 (KIM-1) is a proximal tubular injury biomarker for early detection of acute kidney injury (AKI), with variable performance characteristics depending on clinical and population settings.

**Methods:**

Meta-analysis was performed to assess the diagnostic value of urinary KIM-1 in AKI. Relevant studies were searched from MEDLINE, EMBASE, Pubmed, Elsevier Science Direct, Scopus, Web of Science, Google Scholar and Cochrane Library. Meta-analysis methods were used to pool sensitivity and specificity and to construct summary receiver operating characteristic (SROC) curves.

**Results:**

A total of 2979 patients from 11 eligible studies were enrolled in the analysis. Five prospective cohorts, two cross-sectional and four case-control studies were identified for meta-analysis. The estimated sensitivity of urinary KIM-1 for the diagnosis of AKI was 74.0% (95% CI, 61.0%–84.0%), and specificity was 86.0% (95% CI, 74.0%–93.0%). The SROC analysis showed an area under the curve of 0.86(0.83–0.89). Subgroup analysis suggested that population settings and detection time were the key factors affecting the efficiency of KIM-1 for AKI diagnosis.

**Limitation:**

Various population settings, different definition of AKI and Serum creatinine level used as the standard might have influence on AKI diagnosis. The relatively small number of studies and heterogeneity between them also affected the evaluation.

**Conclusion:**

Urinary KIM-1 may be a promising biomarker for early detection of AKI with considerable predictive value, especially for cardiac surgery patients, and its potential value needs to be validated in large studies and across a broader scope of clinical settings.

## Introduction

Acute kidney injury (AKI) is a common and serious condition recognized in nearly all fields of medical practice. It is characterized as a rapid and intensive decline of renal function associated with series of clinical syndrome which account for high morbidity and mortality [1], [2]. The latest survey reported that almost 2 million people died of AKI every year and the survivors had an enhanced risk of chronic kidney disease [3]. Early diagnosis and intervention of AKI could effectively prevent the occurrence of the outcome. Despite the advanced progress made in etiology and pathology of AKI, the clinical detection and diagnosis was still in controversy. Nowadays, the most widely used and commonly accepted clinical standard for the definition and diagnosis of AKI usually relied on the increase of serum creatinine or decrease of urine output which was proposed by both AKIN (acute kidney injury network) and RIFLE (risk, injury, failure, loss, and ESRD) [4]. Unfortunately, due to the poor sensitivity and specificity and 48 h–72 h time needs, serum creatinine was incapable to comprehensively reflect the time and type of renal injury. Moreover, serum creatinine was also affected by some other factors, such as age, acute and chronic renal failure [5]. These studies suggested that more accurate and efficient measure for AKI diagnosis was urgently required [6]. Lines of evidence showed that urinary NGAL, IL-18, Cys-C, KIM-1 and some other candidate molecules were believed as potential markers to diagnosis of AKI [7], [8]. But until now, none of them are currently established well enough to replace serum creatinine as a marker of renal function. Among various kinds of these markers, growing evidence showed that KIM-1 performed significantly superiority in early detection of AKI than others, especially within 24 hours, well before serum creatinine increase, which made it possible to conduct prevention or treatment strategies at a very early stage of AKI [9], [10].

KIM-1, a type-1 transmembrane protein, was originally found as a putative epithelial cell adhesive molecule containing a novel immunoglobulin domain, which was absent in normal condition but elevated in the proximal tubule apical membrane cells after injury [11], [12], [13]. Previous reports had proved Kim-1 in rat model as an outstanding indicator of kidney injury better than serum creatinine to predict proximal tubule injury [13]. Urinary KIM-1 levels are strongly related to tubular KIM-1 expression in experimental and in human renal disease [12]. Studies in human also indicated that urinary KIM-1 was sensitive and specific marker of injury as well as predictors of outcome [14]. Recently, two systematic reviews had been reported that KIM-1 was an efficient novel urinary biomarker in diagnosis of AKI within 24 hours after kidney injury [15], [16], especially in the diagnosis of ischemic ATN [15]. Although the extensive analyses have been carried out, owing to the limitation of relatively small population settings, heterogeneous patient type, less clinical trial and different detection time, the application of KIM-1 in early diagnosis of AKI still needs to be validated and thoroughly investigated in larger studies. Moreover, since adults are a different population to the children and rare studies were involved in evaluating age effect on urinary KIM-1 level, age might be an important influencing factor which needs to be studied.

To fully understand the diagnostic and predictive performance of urinary KIM-1 of AKI, we conducted a meta-analysis based on 11 original articles, which will be helpful for evaluate their roles on early clinical detection and diagnosis of AKI.

## Methods

### Data Sources and Search Strategy

This meta-analysis was performed in accordance with the Quality of Reporting of Meta-analysis (QUOROM) consensus guidelines and according to a protocol that pre-specified outcomes, search strategies, inclusion criteria, and statistical analysis [17]. Studies were identified by a literature search of the PubMed, MEDLINE, ISI Web of Science, EMBASE, Google Scholar, Scopus, Science direct and Cochrane library up to June 2013 with the following key words: “kidney injury molecule 1” or “KIM-1” plus “acute kidney injury” or “acute renal failure”, without language restriction. Besides, we checked the reference lists of retrieved articles to identify additional studies.The searches were performed independently by 2 investigators (Shao X and Tian L).

### Study Selection

We chose articles and conference papers that had a prospective design or case-control design or cross-sectional design and explored the performance of urinary KIM-1 for the detection of AKI without language or sample size restrictions. Two reviewers (Shao X and Tian L) used the EndNote bibliography manager to check the titles and abstracts of all citations and then retrieved and rescreened full-text articles. The reference lists of reviewed full-text articles were checked for fear losing additional relevant studies.

### Data extraction and quality assessment

Data were extracted by two authors (Shao X and Tian L). From each study, the following information was received: first author, country of origin, year of publication, study design, sample size, population setting (patients after cardiopulmonary bypass surgery, patients after cardiac catheterization, patients admitted to the intensive care unit and patients admitted to emergency department), and patient characteristics (age, sex, and baseline serum creatinine), as well as definition of AKI and number of patients who developed AKI. In addition, data extraction regarding KIM-1 including the laboratory assay used, the reported biomarker value unit (ng per milliliter vs ng per milligram of creatinine), and the timing of the measurement. In relation to the outcomes of interest, the optimal cutoff thresholds, as defined by the authors of the individual studies, the area under the curve (AUC) for the receiver operating characteristic (ROC) and the true-positive, true-negative, false-positive, and false-negative values were recorded.

The methodological quality of studies was individually evaluated by two authors (Shao X and Tian L, two doctors in department of nephrology and were systematically trained for meta-analysis) pivoting on the Quality Assessment of Diagnostic Accuracy Studies (QUADAS) instrument [18], a quality assessment tool specifically developed for systematic reviews of diagnostic accuracy studies to assess bias in the study [19], including 14 questions (each of which is scored as yes, no, or unclear).

### Data Synthesis and Analysis

We conducted STATA version 12 and Meta-Disc to analysis the data [20]. Summary sensitivities, specificities, positive and negative likelihood ratio and diagnostic odds ratios (DOR) with their 95% confidence intervals (95% CIs) were obtained using random effect models with DerSimonian Laird methods or fixed effect models depending on the level of heterogeneity of the study group [21]. Forest plots of sensitivities, specificities and DORs were presented. Moreover, AUC-ROC values with 95% CI were combined. An AUC-ROC>0.70 defines a useful risk predictor [22].

Heterogeneity in meta-analysis indicates the degree of variability in results across studies. It was appraised using Q test P value and the I^2^ index which revealed thresholds for low (25%–49%), moderate (50%–74%), and high (>75%) values [23]. When substantial heterogeneity was found to be present (I^2^>50%), There were three strategies used to assess possible heterogeneity: Spearman correlation coefficient test which can reveal the presence of threshold effect (differences in sensitivities and specificities occurring because of different cut-offs used in different studies to define a positive test result), subgroup analysis and summary ROC analysis [23].

In addition, we used a funnel plot of effect size against its SE to evaluate the publication bias [24]. The funnel plot should be asymmetric when there is publication bias and symmetric in the case of on publication bias. Since the funnel plot approach is limited by the requirement for a range of studies with varying size, we adopted Egger's linear regression test, which measures the funnel plot asymmetry on the natural logarithm scale of DOR (p<0.05 was believed representative of statistically significant publication bias).

## Results

### Search Results and Study Characteristics

**Figure 1:**
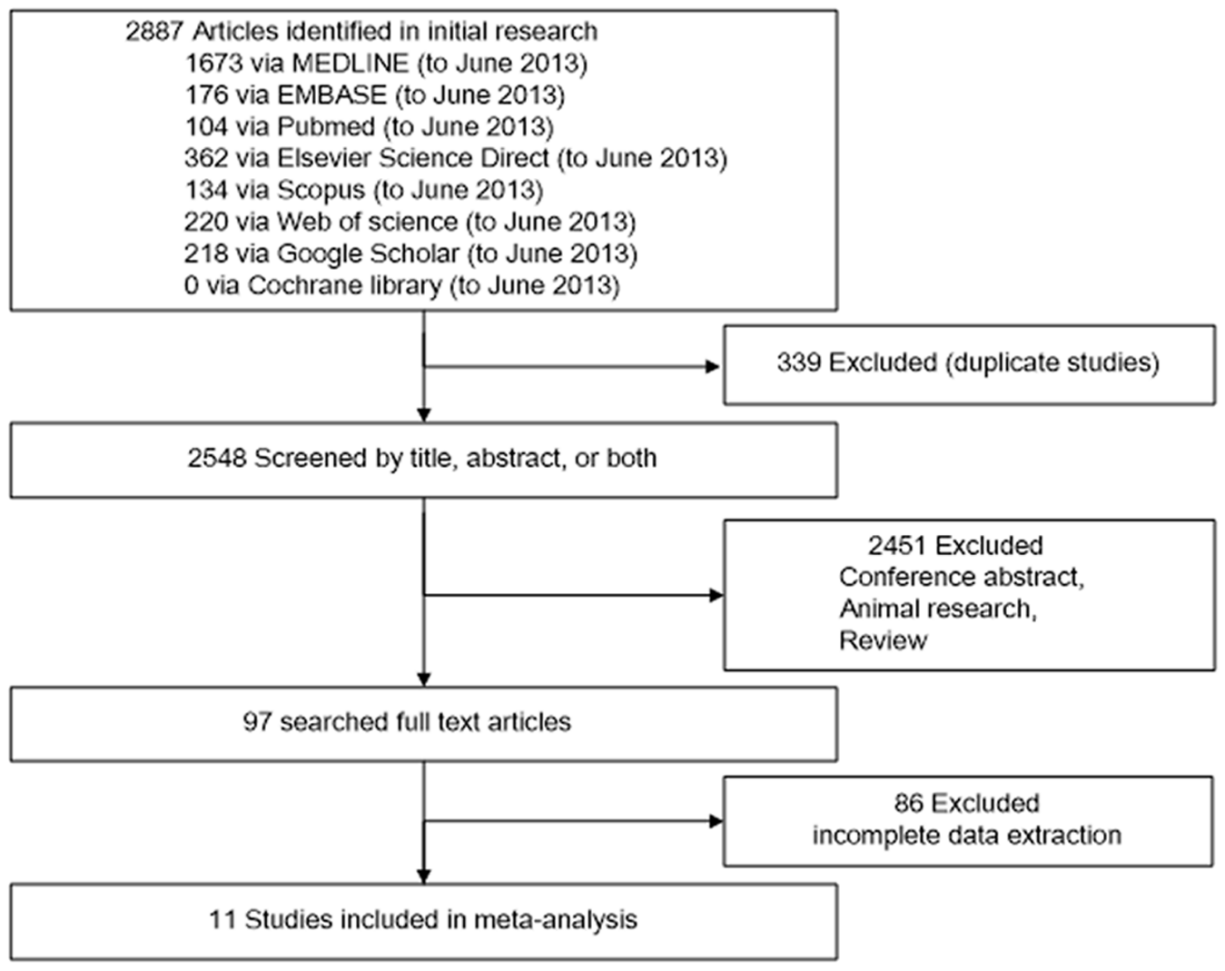
Flow diagram for the review process and outcomes of inclusion and exclusion.

The primary search revealed 2887 publications from variable databases. Firstly, 339 repeated studies were rejected in our research. Then the majority was sifted out based on titles or abstracts. There were 97 articles evaluated in detail. Finally, 11 studies were accepted [9], [10], [25], [26], [27], [28], [29], [30], [31], [32], [33] (Figure 1). Characteristics of the individual studies are listed in Table 1. There were 5 prospective cohort studies, 4 case-control studies and 2 cross-sectional studies adopted in this meta-analysis. Among these studies, Han [10], Genc [32] and Sarafidis [33] studies enrolled children or infants and others all enrolled adults [10], [32], [33]. All these studies were published from 2008 to 2013, varied in sample size (from 40 to 1635), and involved patients in different clinical settings. Four studies focused on patients underwent cardiopulmonary bypass surgery, and the other included cardiac catheterization, ICU patients, emergency department patients and critically ill patients. All samples are mentioned to store at −80°C and blinding of investigators was documented in seven studies with four articles not known. As listed in Table 1, variable definitions of AKI were adopted in the individual studies. Urinary KIM-1 level was measured in all studies by a commercial enzyme-linked immune sorbent assay (ELISA) or microbead-based ELISA and micro-sphere-based Luminex xMAP technology.

**Table 1:** Characteristics of Studies Included in the Meta-analysis.

Study	Country	Design	N	Patients with AKI	Population settings	Age (y)	Males	Baseline Scr (mg/dL)	Definition of AKI	Blinding of investigators
Genc (2013)[32]	Turkey	Prospective cohort study	48	18	NICU	29.9bw	27	1.01a	Scr levels after hour 60 of life >1.3 mg/dL or an increase inScr by either >0.3 mg/dL or an increase of ≥50% from baseline	NR
Nickolas (2012)[31]	USA	A Multicenter Prospective Cohort Study	1635	96	Emergency department	64.4	855	0.9	≥50% increase in SCr more than 3 days and patients exposed to stimuli	YES
Naggar (2012)[27]	Egypt	case-control study	40	21	Critically ill patients	51.75a	16	0.9a	RIFLE criteria	NR
Sarafidis (2012)[33]	Greece	case-control study	35	8	NICU	38.3bw	21	1.13a	sCr ≥1.5 mg/dl for >24 h or rising values >0.3 mg/dl from day of life 1	YES
Endre (2011)[30]	New Zealand Australia USA	Prospective observational study	528	147	patients in ICU	60	210	1.0	≥50% or 0.3mg/dL above baseline pCr of the first sample in the ICU	YES
Ferguson (2010)[28]	USA	cross-sectional study	134	92	General hospital ward; critical care setting; Precatheterization	62.6a	81	NR	≥50% increase in SCr	NR
Liang (2010)[29]	China	case-control study	122	30	CPB surgery	30a	NR	1.01a	RIFLE criteria	YES
Liangos (2009)[9]	USA	Prospective cohort study	103	13	CPB surgery	68	74	1.1	≥50% increase in SCr within 72 h	YES
Han (2009)[26]	USA	prospective cohort study	90	36	CPB surgery	63.6a	61	1.04a	increase in Scr of ≥0.3 mg/dl or 2- to 3-fold within 72 h	YES
Han (2008)[10]	USA	case-control study	40	20	CPB surgery	3.2a	60	0.425a	≥50% increase in SCr	YES
Vaidya (2008)[25]	USA	a cross-sectional study	204	102	General hospital ward; critical care setting; cardiac catheterization	56.9a	102	AKI 1.7–10.0 non-AKI 0.4–1.4	≥50% increase in SCr	NR

Abbreviations: AKI, acute kidney injury; CPB, cardiopulmonary bypass; CS, cardiac surgery; SCr, serum creatinine; ICU, intensive care unit; NR, not reported; NICU, neonatal intensive care unit; KIM-1, kidney injury molecule 1; RIFLE, risk, injury, failure, loss, end-stage renal disease; USA, United States of America.

^a^ Mean baseline SCr level (mg/dL) or age (y,year).

^b^ Gestational age (w,week).

### Quality Assessment

Spearman correlation coefficient of these 11 articles was 0.082 (P = 0.811), suggesting there was no significant threshold effect. The methodological quality of the studies according to the QUADAS tool is summarized in Table S1 (provided as supplementary material). Egger test showed p = 0.003, suggesting there was publication bias. Index test results were interpreted with knowledge of the results of the reference standard for the clinical diagnosis of AKI (based on serum creatinine).

### Data synthesis

**Figure 2:**
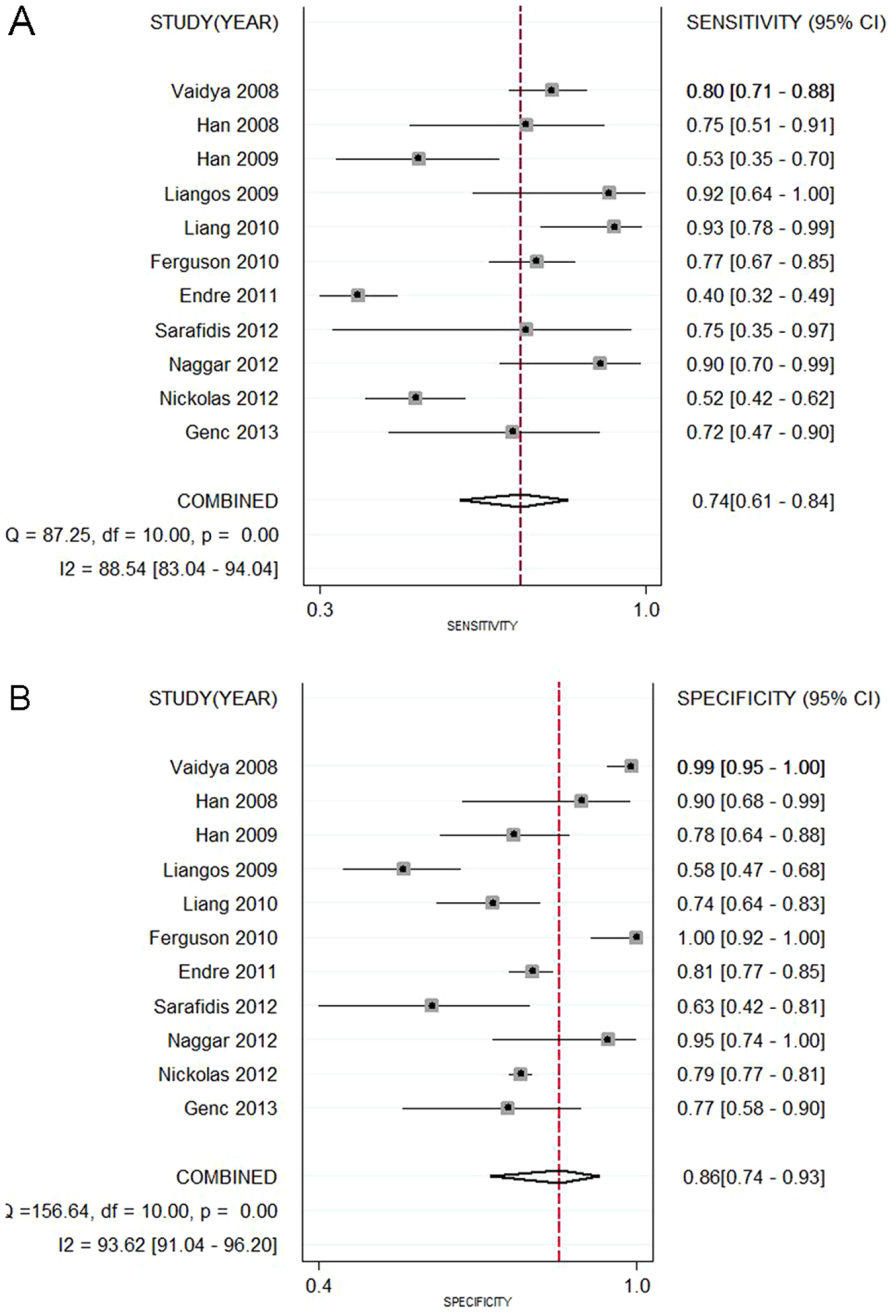
Forest plots of the pooled sensitivity (A) and specificity (B) of urine kidney injury molecule 1 level in predicting acute kidney injury across all settings. The black squares in the gray squares and the horizontal lines represent the point estimate and 95% confidence interval (CI), respectively. The dotted line represents the pooled estimate, and the diamond shape represents the 95% CI of the pooled estimate.

**Figure 3:**
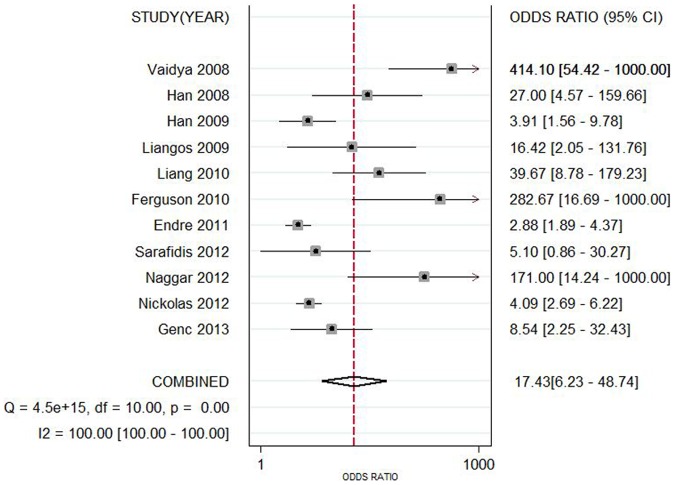
Forest plot of the pooled diagnostic odds ratio of urine kidney injury molecule 1 level in predicting acute kidney injury across all settings. The black squares in the graysquares and the horizontal lines representthe point estimate and 95% confidence interval(CI), respectively. The dotted line represents the pooled estimate, and the diamond shape represents the 95% CI of the pooled estimate.

**Figure 4:**
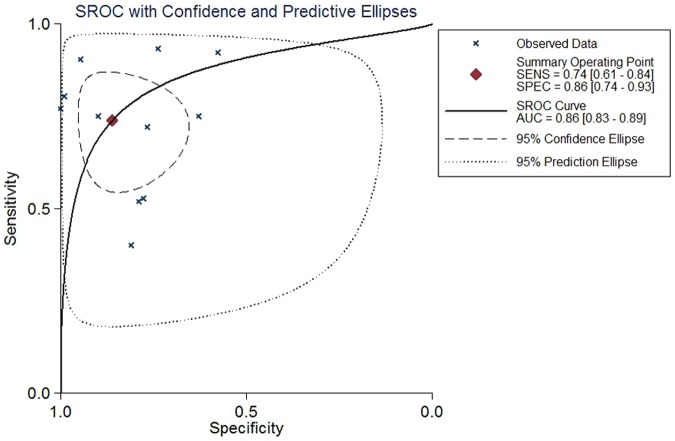
Hierarchical summary receiver perating characteristic (SROC) plots of urine kidney injury molecule 1 level to predict acute kidney injury across all settings. The curve is represented by the straight line; each of the analyzed studies is represented by a circle; the point estimate to which summary sensitivity (SENS) and specificity (SPEC) correspond is represented by the diamond shape, and the respective 95% confidence intervals, by the dashed line, whereas the 95% confidence area in which a new study will be located is represented by the dotted line. Abbreviation: AUC, area under the curve.

**Figure 5:**
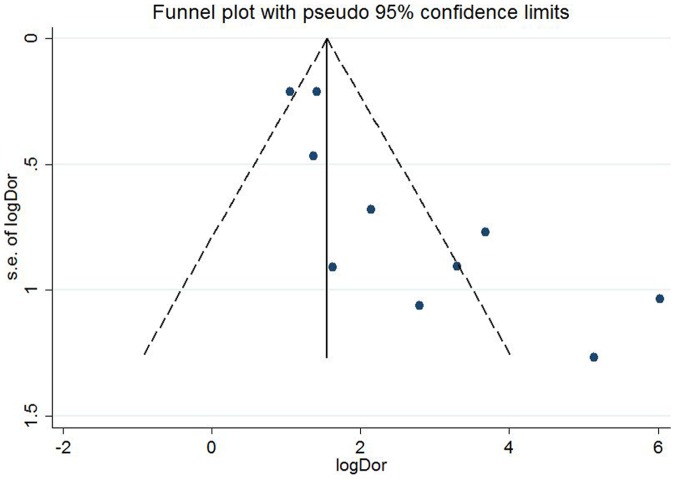
Funnel plot for the evaluation of potential publication bias in diagnosis of KIM-1 for AKI.

Data in the 11 eligible studies were extracted and showed in Table 2, including true-positive, false-positive, false-negative, and true-negative values; various optimal cutoff values for urinary KIM-1; sensitivities; specificities; AUC-ROC (95% CI); Assess method; time of measurement for the diagnosis of AKI. The estimated sensitivity of urinary KIM-1 for the diagnosis of AKI was 74.0% (95% CI, 61.0%–84.0%), and specificity was 86.0% (95% CI, 74.0%–93.0%), with a DOR of 17.43(95% CI, 6.23–48.74) shown as Figure 2 and Figure 3. There was strong heterogeneity both in sensitivity and specificity between studies as evidenced by an I^2^ indexes of 88.54% (83.04–94.04%) and 93.62% (91.04–96.20%) respectively. SROC results showed AUC of urinary KIM-1 was 0.86(0.83–0.89), suggesting that efficiency of KIM-1 for AKI diagnosis was considerable (Figure 4). Funnel plots showed there was publication bias with significant difference (Figure 5).

**Table 2:** Performance of Urinary KIM-1 for AKI diagnosis in Studies Included in the meta-analysis.

study	NO. of patients				Assay method	Timing of measurement	KIM-1 Cutoff	Sensitivity (95%CI)	Specificity (95%CI)	AUC-ROC (95% CI)
	TP	FP	FN	TN						
Genc (2013)[32]	13	7	5	23	ELISA	within day of life 2	≥0.5 ng/mg increase with 3 days	0.733 (NR)	0.769 (NR)	0.791 (NR)
Nickolas (2012)[31]	50	323	46	1216	chemiluminescent microparticle immunoassay	within 12 h after patient enrollment	2.817 ng/ml	0.52 (NR)	0.79 (NR)	0.71 (0.65–0.76)
Naggar (2012)[27]	19	1	2	18	ELISA	within 1 day after patient enrollment	NR	0.909 (NR)	0.9254 (NR)	NR
Sarafidis (2012)[33]	6	10	2	17	ELISA	within day of life 1	0.928 ng/mg	0.8 (NR)	0.625 (NR)	0.608 (NR)
Endre (2011)[30]	59	72	88	309	microsphere-based Luminex xMAP technology	on entry to ICU	1.86 ng/mg	0.40 (0.32–0.48)	0.81 (0.77–0.85)	0.66 (0.61–0.72)
Ferguson (2010)[28]	71	0	21	42	microbead-based sandwich ELISA	NR	1.7 ng/mg	0.77 (0.67–0.85)	1 (0.92–1)	0.89 (0.82–0.94)
Liang (2010)[29]	28	24	2	68	ELISA	6 h post-CPB	1.5 ng/mg	0.933 (NR)	0.739 (NR)	0.881 (0.810–0.933)
Liangos (2009)[9]	12	38	1	52	ELISA	2 h post-CPB	0.42 ng/mg	0.92 (NR)	0.58 (NR)	0.78 (0.64–0.91)
Han (2009)[26]	19	12	17	42	ELISA	post-operation immediately	1.2 ng/mg	0.5143 (NR)	0.7778 (NR)	0.68(0.58–0.78)
Han (2008)[10]	15	2	5	18	ELISA	12 h post-CPB	2.0 ng/mg	0.74 (NR)	0.9 (NR)	0.83 (0.67–0.96)
Vaidya (2008)[25]	82	1	20	101	microbead-based assay	NR	1.73 ng/mg	0.8 (NR)	0.99 (NR)	0.93 (NR)

Abbreviations: AKI, acute kidney injury; AUROC, area under the receiver operating characteristic curve; CI, confidence interval; CPB, cardiopulmonary bypass; FN, false negative; FP, false positive; ICU, intensive care unit; KIM-1, kidney injury molecule 1; NR, not reported; TN, true negative; TP, true positive; ELISA, enzyme-linked immunosorbent assay.

We also performed subgroup analysis by population settings, study design, age, measurement method, and blinding or not. Data showed that consistency of non-prospective studies had significantly been decreased, which because half of them enrolled established AKI patients (Table 3). Detection by ELISA was much more sensitive than no-ELISA method with significantly decreased consistency coefficient, as shown in Table 3. Moreover, age could also affect the consistency, studies in infants or children showed only 23.8% of I^2^ index (Table 3). With regard to the patient population settings, patient underwent CPB showed remarkably increased sensitivity compared with ICU and other kinds of patients. Three studies tested KIM-1 between 2 h to 12 h after CPB, except one immediately after CPB. The sensitivity of combined three studies was 87.0% (77.0%–94.0%), and specificity was 68.0% (61.0%–75%), as shown in Table 4. There was moderate heterogeneity between studies as evidenced by an I^2^ index of 46.2% and Q test P = 0.1559, indicating right detection time was the most important factor affecting diagnosis of AKI (Figure 4).

**Table 3:** Subgroup analysis based on different standard.

Studies		Sensitivity(95%CI)	Specificity(95%CI)	+LR(95%CI)	−LR(95%CI)	DOR(95%CI)	AUC
All studies(11)		0.74(0.61–0.84)	0.86(0.74–0.93)	5.29(2.59–10.79)	0.30(0.19–0.48)	17.43(6.23–48.74)	0.86
I-square(%)		88.54%	93.62%	85.26%	91.08%	100%	
Patient population	Cardiac surgery(4)	0.75(0.65–0.83)	0.70(0.64–0.76)	2.88(1.91–4.35)	0.25(0.08–0.72)	14.4(3.95–52.54)	0.8512
	I-square(%)	83.20%	76.90%	64.30%	82.10%	66.20%	
	ICU and others(7)	0.62(0.57–0.66)	0.81(0.79–0.82)	4.31(2.19–8.48)	0.35(0.21–0.57)	16.15(5.41–48.20)	0.7703
	I-square(%)	91.30%	90.70%	88.70%	92.20%	88.10%	
Study design	prospective(5)	0.49(0.44–0.55)	0.78(0.77–0.80)	2.33(2.03–2.69)	0.62(0.49–0.77)	3.86(2.72–5.47)	0.7947
	I-square(%)	80.60%	81.20%	0.00%	55.90%	22.20%	
	non-prospective(6)	0.81(0.76–0.85)	0.87(0.83–0.91)	10.41(2.44–45.00)	0.21(0.17–0.28)	59.05(15.02–232)	0.9229
	I-square(%)	24.90%	91.00%	92.10%	2.70%	64.70%	
Age	Adults(8)	0.63(0.58–0.67)	0.79(0.78–0.81)	2.96(1.95–4.49)	0.36(0.23–0.57)	12.44(4.87–31.55)	0.8459
	I-square(%)	91.50%	91.80%	86.00%	91.40%	86.40%	
	Infants or chidren(3)	0.8(0.67–0.89)	0.86(0.75–0.93)	5.86(1.97–417.41)	0.26(0.14–0.48)	25.99(5.37–125.8)	0.8465
	I-square(%)	23.80%	45.50%	56.00%	30.10%	56.10%	
Measurement method of KIM-1	ELISA(8)	0.77(0.71–0.82)	0.75(0.70–0.79)	3.53(2.05–6.08)	0.27(0.16–0.45)	18.79(6.74–52.39)	0.8807
	I-square(%)	66.60%	85.20%	81.50%	71.20%	66.50%	
	non-ELISA(3)	0.55(0.50–0.61)	0.80(0.79–0.82)	3.65(1.59–8.39)	0.46(0.25–0.83)	9.30(2.50–34.58)	0.1599
	I-square(%)	95.30%	94.70%	92.40%	95.20%	92.70%	
Blinding or not	Blinding(7)	0.54(0.49–0.59)	0.78(0.76–0.80)	2.48(2.09–2.96)	0.5(0.35–0.69)	6.13(3.34–11.28)	0.8102
	I-square(%)	87.80%	78.10%	33.40%	75.10%	66.80%	
	non-Blinding(4)	0.79(0.74–0.84)	0.95(0.91–0.98)	20.99(1.51–292.37)	0.22(0.17–0.29)	100.02(10.12–988.14)	0.8813
	I-square(%)	0.00%	85.80%	89.90%	12.30%	79.30%	

Abbreviations: AUC, area under the receiver operating characteristic curve; CI, confidence interval; ICU, intensive care unit; KIM-1, kidney injury molecule 1; ELISA, enzyme-linked immunosorbent assay; +LR, positive likelihood ratio; -LR, negative likelihood ratio; DOR, diagnositic odds ratio.

**Table 4:** Subgroup analysis based on patient type and detection time.

Studies		Sensitivity(95%CI)	Specificity (95%CI)	LR+(95%CI)	LR-(95%CI)	DOR(95%CI)	AUC
All studies(11)		0.74(0.61–0.84)	0.86(0.74–0.93)	5.29(2.59–10.79)	0.30(0.19–0.48)	17.43(6.23–48.7)	0.86
I-square(%)		88.54%	93.62%	85.26%	91.08%	100%	
Patient population	Cardiac surgery(4)	0.75(0.65–0.83)	0.70(0.64–0.76)	2.88(1.91–4.35)	0.25(0.08–0.72)	14.4(3.95–52.54)	0.8512
	I-square(%)	83.20%	76.90%	64.30%	82.10%	66.20%	
	2h after surgery(3)	0.87(0.77–0.94)	0.68(0.61–0.75)	3.21(1.75–5.90)	0.18(0.07–0.42)	28.53(10.43–78.07)	0.9109
	I-square(%)	46.20%	82.00%	79.80%	32.50%	0.00%	

Abbreviations: AUC, area under the receiver operating characteristic curve; CI, confidence interval; +LR, positive likelihood ratio; -LR, negative likelihood ratio; DOR, diagnositic odds ratio.

## Discussion

Recently, the serum creatinine, common standard to test AKI, displayed numerous limitations which affect the early diagnosis and prognosis of AKI [6]. In order to enhance the ability to predict the occurrence of AKI and facilitate timely introduction of AKI-specific therapies, more and more effort were made to discover novel urinary biomarkers prior to serum creatinine [6].

In the present meta-analysis, we generalized all published studies which have examined the performance characteristics of one of the urinary biomarker, KIM-1, to fully evaluate the diagnostic value. Of all the identified cohort studies of patients at risk for AKI, 11 could be meta-analyzed for the diagnosis of AKI, whereby urinary KIM-1 showed good performance characteristics with high sensitivity and specificity, especially in patients undergoing cardiac surgery.

To our knowledge, it might be a novel meta-analysis which assessed the diagnostic value of KIM-1 for AKI. Interestingly, pooled analysis of the studies exhibited relatively much more sensitive role in predicting AKI, with combined 74.0% sensitivity and 86.0% specificity. However, the inconsistency factor was very huge. Accordingly, the source of heterogeneity present in this analysis on AKI early detection was analyzed using subgroup analysis by study design, population settings, time point of measurements of patients and other factors. The results showed that age, population settings and time of measurement of KIM-1 were the main source of heterogeneity. Furthermore, subgroup analysis by other factors did not expressively alter the diagnostic significance of KIM-1.

Urinary KIM-1 was reported to be affected by detection time [34]. As shown in this study, only 50% sensitivity was shown immediately after CPB [26]. Two studies showed more than 90% sensitivity when KIM-1 was tested 2 h and 6 h after CPB [9], [29]. However, the sensitivity was decreased to 74% 12 h after CPB [10]. These results suggested that right time adoption could remarkably increase the success of AKI diagnosis. More studies with a larger sample size are thus needed to further elucidate the diagnostic value of KIM-1 for AKI.

Urinary KIM-1 level also has been shown to correlate with fibrotic changes in experimental models of chronic kidney disease [35]. Seven studies included in this paper were involved in ICU patients, Emergency Department patients and general hospital ward, which might combined some chronic disease or other unknown diseases. Owing to the possibly fundamental expression of KIM-1, the elevation of KIM-1 in AKI detection might be affected to some extent. However, we cannot completely rule out other possibility that some other covariates might potentially account for part of the heterogeneity. Furthermore, patients with AKI in ICU, Emergency Department or general hospital ward had distinct causes including ischemia, sepsis, contrast media and renal toxin, which can affect the expression of KIM-1.Yun Huang et al had revealed that urinary KIM-1 level was the highest in ischemic acute tubule necrosis patients [15]. However, due to scant data we cannot assess the performance of KIM-1 in different causes of AKI.

On the basis of age subgroup analysis, we detected that urinary KIM-1 had better diagnostic accuracy in infants or children than in adults. We deduced the significant comorbid conditions, such as hypertension, diabetes mellitus and atherosclerotic, are more prevailing in adults and may influence urinary KIM-1 concentrations. Therefore, the performance of urinary KIM-1 for AKI in infants/children may be more reliable than in adults. In addition, the measurement of KIM-1 needs standardization as a series of independently assays have been used. The chief methodology of KIM-1 measurement is based on ELISA, however, varible antibodies, reagents and reaction designs lead to a difference of KIM-1 test performance which leads to difficulties in data comparison.

Although we examined the diagnostic value of urinary KIM-1 level as a predictor of early AKI, we were unable to assess whether this marker adds value to other clinical factors or to a panel of urinary markers of kidney injury, which might be a limitation.

Furthermore, the limitations of this meta-analysis cannot be ignored. Limitations of the analysis include the small number of studies, heterogeneity in study populations with a broad range of clinical settings, variable definitions of AKI (reference standard test), variable biomarker cutoff values (index test), variable KIM-1 assays and variable duration of follow-up. One additional important limitation is the current AKI vriteria using serum creatinine as a diagnositc index, which cannot reveal subclinical kidney injury and might underscore the specificity of KIM-1. Moreover, a certain degree of publication bias was found in the analysis concerning KIM-1 for AKI diagnosis according to Egger test and funnel plot. The DOR estimate in our meta-analysis might be overestimated because of publication and reporting bias.

Recently, more and more experts suggested that combination of multiple biomarkers to form a biomarkers panel was an optimal way to detect AKI more efficiently and accurately [36], [37]. One study enrolled in this meta-analysis also reported on the combination application of the urinary biomarkers KIM-1 and interleukin-18 for early detection of AKI [29]. For early predicting progressive AKI, the estimated sensitivity was 81.8% and specificity was 83.8%.

In conclusion, the present meta-analysis demonstrates that measurement of urinary KIM-1, a proximal tubular injury marker, appears to be a relatively good discrimination for the diagnosis of AKI in hospital-based cohorts of patients at risk of AKI, especially in patients underwent cardiac surgery after CPB 2 h to 12 h, suggesting KIM-1 might be a specific predictor for early AKI. The potential diagnostic and prognostic value of this biomarker needs to be validated further in large cohort studies and clinical settings.

## Supporting Information

Table S1 **Methodological quality of the 11 studies included in the meta-analysis.**
(DOC)

Checklist S1 **PRISMA statement of the meta-analysis.**
(DOC)

Diagram S1 **Four phase Flow diagram of the meta-analysis.**
(DOC)
