# Supplementary material for: Diagnostic Value of Urinary Kidney Injury Molecule 1 for Acute Kidney Injury: A Meta-Analysis
Source: PLoS One. 2014 Jan 3;9(1):e84131. doi: 10.1371/journal.pone.0084131 (PMC3880280; doi:10.1371/journal.pone.0084131)
Supplement: Table S1 — Methodological quality of the 11 studies included in the meta-analysis. (DOC) [file pone.0084131.s001.doc]

Table S1 Methodological quality of the 11 studies included in the meta-analysis.

| Study | Item | | | | | | | | | | | | | | Total(/14) |
| --- | --- | --- | --- | --- | --- | --- | --- | --- | --- | --- | --- | --- | --- | --- | --- |
|  | 1 | 2 | 3 | 4 | 5 | 6 | 7 | 8 | 9 | 10 | 11 | 12 | 13 | 14 |  |
| Genc (2013)[32] | N | Y | Y | Y | Y | N | Y | Y | Y | Y | Y | Y | Y | Y | 12 |
| Nickolas (2012)[31] | U | U | Y | Y | Y | Y | Y | Y | Y | Y | Y | Y | Y | Y | 12 |
| Naggar (2012)[27] | N | N | Y | Y | Y | Y | Y | Y | N | N | Y | Y | Y | Y | 10 |
| Sarafidis (2012)[33] | N | Y | Y | Y | Y | Y | Y | Y | Y | Y | Y | Y | Y | Y | 13 |
| Endre (2011)[30] | Y | Y | Y | Y | Y | N | Y | Y | Y | Y | Y | Y | N | Y | 12 |
| Ferguson (2010)[28] | N | Y | Y | Y | Y | Y | Y | Y | N | U | Y | Y | Y | Y | 11 |
| Liang (2010)[29] | Y | Y | Y | Y | Y | N | Y | Y | N | Y | Y | Y | Y | Y | 12 |
| Liangos (2009)[9] | Y | Y | Y | Y | Y | N | Y | Y | N | Y | Y | Y | Y | Y | 12 |
| Han (2009)[26] | Y | Y | Y | Y | Y | N | Y | Y | Y | Y | Y | Y | Y | Y | 13 |
| Han (2008)[10] | Y | Y | Y | Y | Y | Y | Y | Y | Y | Y | Y | Y | Y | Y | 14 |
| Vaidya (2008)[25] | N | Y | Y | Y | N | Y | Y | Y | N | U | Y | Y | Y | Y | 10 |

1. Was the spectrum of the study participants representative of the patients who will receive the test in practice?

2. Were selection criteria clearly described?

3. Was the reference standard* likely to classify the target condition correctly?

4. Was the period between performance of the reference standard and the index test short enough to be reasonably sure that the target condition did not change between the two tests?

5. Did the whole sample or a random selection of the sample receive verification using the reference standard?

6. Did participants receive the same reference standard regardless of the index test result?

7. Was the reference standard independent of the index test?

8. Was the execution of the index test described in sufficient detail to permit its replication?

9. Was the execution of the reference standard described in sufficient detail to permit its replication?

10. Were the index test results interpreted without knowledge of the results of the reference standard?

11. Were the reference standard results interpreted without knowledge of the results of the index test?

12. Were the same clinical data available when the test results were interpreted as would be available when the test is used in practice?

13. Were uninterpretable, indeterminate or intermediate test results reported?

14. Were withdrawals from the study explained?

* The reference standard refers to the clinical diagnosis of acute kidney injury based on serum creatinine. (code item: Y = Yes; N = No; U = unclear)
