# Supplementary material for: Diagnostic Value of Urinary Kidney Injury Molecule 1 for Acute Kidney Injury: A Meta-Analysis
Source: PLoS One. 2014 Jan 3;9(1):e84131. doi: 10.1371/journal.pone.0084131 (PMC3880280; doi:10.1371/journal.pone.0084131)
Supplement: Diagram S1 — Four phase Flow diagram of the meta-analysis. (DOC) [file pone.0084131.s003.doc]

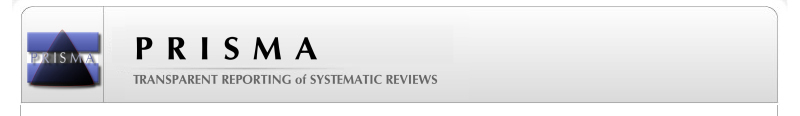
**PRISMA 2009 Flow Diagram**

**Screening**

**Included**

**Eligibility**

**Identification**

Records identified through database searching
(n = 2887 )

Additional records identified through other sources
(n = 0 )

Records after duplicates removed
(n = 2548 )

Records screened
(n = 2548 )

Records excluded
(n = 2451 )

Full-text articles assessed for eligibility
(n = 97 )

Full-text articles excluded, with reasons
(n = 86 )

Studies included in qualitative synthesis
(n = 11 )

Studies included in quantitative synthesis (meta-analysis)
(n = 11 )
